# Editorial: Transcription factors and arrhythmogenesis

**DOI:** 10.3389/fphys.2023.1169747

**Published:** 2023-02-28

**Authors:** Yu-Hsun Kao, Yi-Jen Chen, Satoshi Higa, Nipon Chattipakorn, Gaetano Santulli

**Affiliations:** ^1^ Graduate Institute of Clinical Medicine, College of Medicine, Taipei Medical University, Taipei, Taiwan; ^2^ Department of Medical Education and Research, Wan Fang Hospital, Taipei Medical University, Taipei, Taiwan; ^3^ Department of Internal Medicine, Division of Cardiovascular Medicine, Wan Fang Hospital, Taipei Medical University, Taipei, Taiwan; ^4^ Cardiac Electrophysiology and Pacing Laboratory, Division of Cardiovascular Medicine, Makiminato Central Hospital, Urasoe, Japan; ^5^ Cardiac Electrophysiology Research and Training Center, Faculty of Medicine, Chiang Mai University, Chiang Mai, Thailand; ^6^ Department of Medicine, Wilf Family Cardiovascular Research Institute, Fleischer Institute for Diabetes and Metabolism (FIDAM), Albert Einstein College of Medicine, New York, NY, United States; ^7^ Einstein-Mount Sinai Diabetes Research Center (ES-DRC), Department of Molecular Pharmacology, Einstein Institute for Aging Research, Institute for Neuroimmunology and Inflammation (INI), Albert Einstein College of Medicine, New York, NY, United States

**Keywords:** cardiovascular medicine, induced pluripotent stem cell (iPSC), *LMNA*, *ZFHX3*, bioengineering, cardiac arrhythmia, PKCε

The present Research Topic, entitled “Transcription Factors and Arrhythmogenesis” aims at highlighting the functional role of gene regulatory networks of cardiac enriched transcription factors and their target genes in the pathophysiology of cardiac arrhythmias. Generally speaking, transcription factors bind to the promoter regions of target genes and regulate their expression (Ciccarelli et al., 2011; Larsen et al., 2020; Henley and Koehler, 2021; Tang et al., 2021; de Mattos et al., 2022; Xiao et al., 2022; Jiang and Qian, 2023; Yu et al., 2023; Zhao et al., 2023). Transcription factors can regulate arrhythmogenesis by modulating the expression of genes involved in the cardiac conduction system and/or myocardial structures, as well as through indirect effects on ionic currents, calcium homeostasis, inflammation, oxidative stress, and cardiac remodeling (Liang et al., 2015; Lu et al., 2015; Crespo-Garcia et al., 2022).

Tom McDonald and collaborators elegantly describe the phenotypic variability in iPSC-induced cardiomyocytes and cardiac fibroblasts carrying diverse mutations in the *LMNA* gene, encoding lamin A/C (Yang et al.). These mutations are known to lead to familial arrhythmogenic cardiomyopathy with a high penetrance; however, there is a phenotypic variability in terms of disease onset, severity, and rate of progression. Recent evidence has shown that induced pluripotent stem cell (iPSC) represent a reliable strategy to create disease-in-a-dish models (Varzideh et al., 2022). Thus, the authors generated seven patient-specific iPSC lines with different *LMNA* mutations and successfully differentiated them in cardiomyocytes and cardiac fibroblasts. They observed electrophysiological aberrations, sarcomere disarray, and increased apoptosis in cardiomyocytes, and detected several irregularities of the nuclear membrane morphology in cardiac fibroblasts. Intriguingly, co-culture assays of cardiomyocytes and fibroblasts carrying *LMNA* mutations show exaggerated electrical disturbances (Yang et al.), suggesting that conduction properties of cardiomyocytes might be adversely affected after coculture with fibroblasts in *LMNA* mutation-associated dilated cardiomyopathy. Additionally, patient- or mutation-specific iPSC may serve as an ideal platform for predicting and testing new effective therapeutics.

The *ZFHX3* gene is one of the most studied genes associated with atrial fibrillation (AFib) (Benjamin et al., 2009; Tomomori et al., 2018; Cheng et al., 2019). In a single-center, retrospective, observational cohort study conducted in 1,782 patients who underwent AFib catheter ablation, Hwang et al. found an association between genetic variants of *ZFHX3* and extra-pulmonary vein (PV) triggers. Extra-PV triggers are main causes in AFib recurrence post catheter ablation. *ZFHX3* knockdown in HL-1 atrial myocytes was found to induce electrical remodeling and increase metabolic stress (Cheng et al., 2019; Lkhagva et al., 2021). These pieces of evidence might explain how extra-PV triggers occur in *ZFHX3* genetic variants after ablation.

Qin et al. examined the effects of Gluconolactone (D-glucono-1,5-lactone, GDL) in cardiac ischemia/reperfusion (I/R) injury both *in vivo* (in mice) and *in vitro* (in neonatal cardiomyocytes). GDL is a food additive (E-number: E575) present in several dietary products including bread, cheese, wine, yogurt, and tofu (Romo-Rodriguez et al., 2015). The authors observed that GDL attenuated I/R injury and reduced reperfusion-induced arrhythmias and oxidative stress. They also provide a mechanism explaining these findings, showing that GDL acts as a potent activator of PKCε-mediated ERK signaling (Qin et al.). PKCε may provide cardioprotection in I/R injury *via* activating mitochondrial ALDH2 to scavenge toxic aldehyde-lipid peroxidation products, opening of mitochondrial K_ATP_ channels to decrease ROS production and calcium overload, and inhibiting the activation of L-type calcium channels (Inagaki et al., 2006; Ferreira et al., 2012). Moreover, although not directly investigated in the Qin’s paper, PKCε is a known activator of the transcription factors c-Jun and STAT3 (signal transducer and activator of transcription 3) (Batarseh et al., 2010; Wang et al., 2018). Accordingly, GDL was suggested to have cardioprotective potential against I/R injury.

In conclusion, the research area investigating how transcription factors are linked to the pathophysiology of cardiac arrhythmias is quite active, and further studies are warranted to identify novel molecular mechanisms and eventually therapeutic targets to tackle this major issue in cardiovascular medicine.
